# China Protocol for early screening, precise diagnosis, and individualized treatment of lung cancer

**DOI:** 10.1038/s41392-025-02256-1

**Published:** 2025-05-27

**Authors:** Chengdi Wang, Bojiang Chen, Shufan Liang, Jun Shao, Jingwei Li, Liuqing Yang, Pengwei Ren, Zhoufeng Wang, Wenxin Luo, Li Zhang, Dan Liu, Weimin Li

**Affiliations:** 1 Department of Pulmonary and Critical Care Medicine, State Key Laboratory of Respiratory Health and Multimorbidity, Targeted Tracer Research and Development Laboratory, Frontiers Science Center for Disease-related Molecular Network, West China Hospital, West China School of Medicine, Sichuan University, Chengdu, China; 2 Frontiers Medical Center, Tianfu Jincheng Laboratory, Chengdu, China

**Keywords:** Lung cancer, Health care

## Abstract

Early screening, diagnosis, and treatment of lung cancer are pivotal in clinical practice since the tumor stage remains the most dominant factor that affects patient survival. Previous initiatives have tried to develop new tools for decision-making of lung cancer. In this study, we proposed the China Protocol, a complete workflow of lung cancer tailored to the Chinese population, which is implemented by steps including early screening by evaluation of risk factors and three-dimensional thin-layer image reconstruction technique for low-dose computed tomography (Tre-LDCT), accurate diagnosis *via* artificial intelligence (AI) and novel biomarkers, and individualized treatment through non-invasive molecule visualization strategies. The application of this protocol has improved the early diagnosis and 5-year survival rates of lung cancer in China. The proportion of early-stage (stage I) lung cancer has increased from 46.3% to 65.6%, along with a 5-year survival rate of 90.4%. Moreover, especially for stage IA1 lung cancer, the diagnosis rate has improved from 16% to 27.9%; meanwhile, the 5-year survival rate of this group achieved 97.5%. Thus, here we defined stage IA1 lung cancer, which cohort benefits significantly from early diagnosis and treatment, as the “ultra-early stage lung cancer”, aiming to provide an intuitive description for more precise management and survival improvement. In the future, we will promote our findings to multicenter remote areas through medical alliances and mobile health services with the desire to move forward the diagnosis and treatment of lung cancer.

## Introduction

Lung cancer is the leading cause of incidence and mortality related to malignancy worldwide, resulting in over 2.48 million new cases and 1.8 million deaths per year.^1^ In China, lung cancer has also emerged as the top formidable health challenge and it is estimated that there are 1.06 million people were diagnosed and 0.73 million people died from lung cancer annually.^2^ The tumor stage serves as the most important factor related to patient survival, as the 5-year survival rate of stage IA patients is about 82%, while the ratio decreases to 7% in stage IVB patients.^3^ Thus, to sufficiently improve the overall outcome of lung cancer, early effective screening, precise diagnosis, and optimal individualized treatment are vitally important.

In early-stage lung cancer, few obvious symptoms can be recognized, leading to diagnosis at advanced stages, which hampers prompt intervention.^4^ Thus, identifying patients at high risk of lung cancer, especially screening in asymptomatic cases, is critical. Two randomized controlled trials, the National Lung Screening Trial (NLST) and Nederlands-Leuvens Longkanker Screenings Onderzoek (NELSON) trial, have provided evidence that low-dose computed tomography (LDCT) screening reduces the mortality of lung cancer.^5,6^ However, along with the increased detection of pulmonary nodules, it’s essential to accurately discriminate the malignant nodules from benign lesions to give timely intervention of lung cancer and reduce the overdiagnosis and overtreatment of benign nodules.^7^ Moreover, current lung cancer screening guidelines indeed decreases the death rate, but the problem of missed diagnosis in specific population caused by international criteria remains to be solved.^8^ Thus, establishing a screening approach suitable for the Chinese population is warranted.^9^ In recent years, artificial intelligence (AI) has developed rapidly, and the advent of large language models (LLMs) represents a new wave in this field, which inspires deeper human-computer interaction.^10–12^ The sustaining enthusiasm of attempting to integrate AI and medicine also provides novel tools for lung cancer care, including screening, diagnosis, prognosis, and so on.^13–15^ Nevertheless, the limited interpretability and generalization may hinder real-world practicality, requiring technological innovation and transformation to improve the usability of AI systems.^16^

**Fig. 1:**
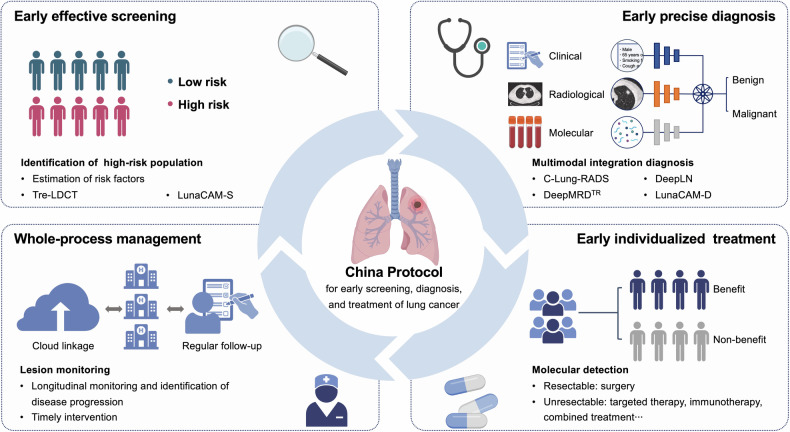
Overview of China Protocol for lung cancer management. The China Protocol consists of screening, diagnosis, treatment, and whole-process management of lung cancer. Through estimation of risk factors, the high-risk cohort can be identified and undergo low-dose computed tomography (LDCT) examination or biomarker-based screening by lung cancer alertness by ctDNA methylation (LunaCAM)-S. Then, the artificial intelligence (AI)-supported systems assist in lung cancer diagnosis, and the diagnostic biomarkers can further improve the accuracy. After diagnosis, the molecular characterization helps to provide precise treatment plans. In addition, the regular follow-up can identify the disease progression and facilitate timely intervention. C-Lung-RADS Chinese Lung Nodules Reporting and Data System, Tre-LDCT three-dimensional thin-layer image reconstruction technique for low-dose computed tomography

With the purpose of improving the detection rate of early-stage lung cancer and assisting optimal intervention, we proposed the China Protocol with realistic applicable potential which is composed of four key steps: screening by early identification of high-risk population and three-dimensional reconstruction CT examination, diagnosis by AI and novel biomarkers, treatment through non-invasive molecular characterization approaches, and whole-process management (Fig. 1). Through the implementation of China Protocol, we aim to diagnose lung cancer in earlier stages, especially in the “ultra-early stage”, which is described first and essentially refers to stage IA1 lung cancer, as well as improving the overall survival.

## Novel effective screening approaches of lung cancer and practice in western China

The determination of the population at high risk of lung cancer faces the challenge of varied definitions in terms of the same risk factors in different regions, leading to potential missed diagnosis by non-customized screening standards.^17^ Therefore, we studied the risk factors in western China population to accurately identify the high-risk group and the results indicated that lung cancer more frequently occurs in the group of age over 40 years old, with smoking history, chronic obstructive pulmonary disease (COPD), diffuse pulmonary fibrosis, and previous history of malignancy.^18^ Then, the selected high-risk patients can undergo the CT examinations. To solve the missed diagnosis of subtle lesions due to the coarse spatial resolution of thick-slice CT, we developed a novel three-dimensional thin-layer image reconstruction technique for LDCT (Tre-LDCT) to effectively detect suspicious lesions and help discover lung cancer. The Tre-LDCT achieved efficient detection of pulmonary nodules with a sensitivity around 96%, along with reconstruction and visualization of the lesions to assist in radiological interpretation.^19^ Subsequently, to further determine the high-risk nodules detected on CT, we meticulously crafted an assessment tool consisting of nine factors, such as age, smoking, family history of malignancy, nodule diameter, spiculation, lobulation, and mixed ground-glass opacity (mGGO) density, were associated with lung cancer.^20^ Moreover, to aid timely and precise lung cancer management, we first defined the stage IA1 lung cancer as “ultra-early stage lung cancer”, a more intuitive description which refers to lung cancer that is in the initial developing stage, with nodules with diameters no more than 1 cm, localized, without lymph node or distant metastasis, and possessing better prognosis. In real-world validation, the thin-slice LDCT has improved lung cancer screening efficiency especially for early-stage (stage I) and ultra-early stage lung cancer.^21^

## Development and application of precise diagnosis approaches for lung cancer

Although the screening approaches have facilitated the detection of pulmonary nodules, it is challenging to make accurate disease determination of the identified nodules, which may result in misdiagnosis. Moreover, the pathological diagnosis relies upon invasive tissue sampling, which is inconvenient and may cause complications.^22^ Thus, previous research has tried to realize non-invasive and automated diagnosis by AI-enabled image decoding.^23,24^ The efficiency of commercial computer-aided detection (CAD) systems has been validated.^25^ We have explored the capability of AI in processing and analyzing multidimensional medical data for information extraction, image standardization, lesion segmentation, representation fusion, and so on.^26–29^ And the technological innovation provides robust tools for lung cancer diagnosis, as elaborated as follows.

Firstly, we have established a respiratory disease database involving lung cancer and other pulmonary diseases, which serves as the foundation of model construction, consisting of radiological images, laboratory test results, and other clinical text data from 434,735 patients.^30,31^ Then, we developed a two-stage segmentation framework to accurately delineate the lung regions and lesions using the backbone of DeepLabv3, obtaining clearer boundaries than human experts.^26^ Subsequently, to capture the radiological characteristics of the segmented pulmonary nodules from CT images, a multi-scale cost-sensitive neural network which ensembled three light models training on CT patches cropped in different sizes was proposed to extract the key features that were of diagnostic values, such as signs of lobulation, spicule, and cavity.^32^ Also, we provided a Semi-Supervised Medical image Detector (SSMD) for effective supervision of unlabeled data, which could reduce the labor of manual annotating and generate standardized labels for model training.^33^ In addition, we innovatively proposed a unified feature fusion method which integrated multimodal data, including demographics, chief complaints, laboratory results, and medical images, to improve the model performance. By leveraging transformer as the backbone and bringing in the bidirectional multimodal attention, the performance of this approach surpassed the image-only and the non-unified methods.^28^

Based on the abovementioned algorithm innovation, we then developed lung cancer diagnosis models. For sub-centimeter pulmonary nodules, baseline plus follow-up lesion analysis achieved a diagnostic area under the curve (AUC) of 0.942 and was comparable to the senior clinician.^34^ Then, for pulmonary nodules detected with more diverse characteristics, we established a multi-dimensional stepwise lung cancer risk assessment model, Chinese Lung Nodules Reporting and Data System (C-Lung-RADS), which stratified the nodules into low-, mid-, high-, and extremely high-risk. And the system demonstrated a higher sensitivity in lung cancer diagnosis, surpassing the current international benchmark Lung CT Screening Reporting and Data System (Lung-RADS) v2022 (87.1% *vs*. 63.3%). Then, personalized plans are made to avoid overdiagnosis of low-risk cases, as well as alleviating missed diagnosis of high-risk population.^35^ We also designed a semi-automated framework, named DeepLN, to identify lung cancer by deep neural networks on CT images, showing an accuracy of 92.46% in discrimination between benign and malignant lesions.^36^ Moreover, to address the diagnostic challenge resulted by the shared radiological abnormalities in different pulmonary diseases, DeepMRD^TR^ was designed to precisely discriminate lung cancer from pneumonia, tuberculosis, interstitial lung disease, and so on.^30^ Importantly, for clinical application, we have developed an AI-powered product for malignancy risk evaluation and diagnosis of pulmonary nodules, and the AI system-assisted approach missed less ultra-early stage lung cancer than manual reading (1.5% *vs.* 10.6%).^37^

## Discovery and validation of biomarkers for early-stage lung cancer

Currently, traditional biomarkers for lung cancer identification, such as carcinoembryonic antigen (CEA), cancer antigen 125 (CA125), and cytokeratin-19 fragment (CYFRA 21-1) are commonly utilized.^38,39^ However, the limited performance in the early-stage lung cancer with sensitivity around 0.6 hindered accurate detection, consequently raising the necessity of exploring novel biomarkers to offer new insights into the early diagnosis of lung cancer.^40^

**Fig. 2:**
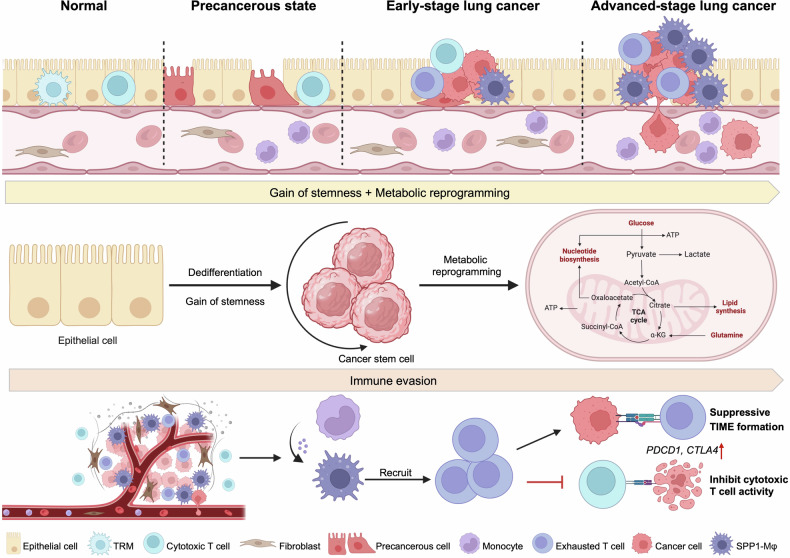
Multi-stage tumorigenesis and progression of lung cancer. The progression of lung cancer is a multifaceted biological process including various stages and steps. During the progression, epithelial cells acquire stemness and undergo metabolic reprogramming. Concurrently, there is a progressive increase in exhausted T cells and SPP1-macrophages. Furthermore, our findings indicate that the multidimensional communication between lung stem cells and immune cells plays a crucial role in sustaining cellular stemness and facilitating tumor immune evasion, thereby promoting the development of lung cancer. TIME, tumor immune microenvironment; TRM, tissue-resident macrophage

Comprehensive deciphering the mechanisms of lung cancer carcinogenesis is crucial to find potential diagnostic targets. The occurrence and development of lung cancer are complex multi-stage processes that may be affected by molecular aberrations of cancer cells and dynamic regulation of tumor microenvironment (TME).^41^ We proposed the “seed-and-soil” hypothesis underpinning lung cancer pathogenesis and found that epithelial cells, such as AT2 and basal cells, serve as the “seeds”. Gradual dedifferentiation and enhanced stemness of these cells are critical steps during lung cancer evolution. And the marker genes, including midkine (*MDK*) and tissue inhibitor matrix metalloproteinase 1 (*TIMP1*), which represent the process of metabolism regulation, are overexpressed, serving as potential biomarkers.^42,43^ Through high-throughput multiomics analysis, we then found that hypomethylation and high expression of *PRAME* can promote the progression of early-stage lung cancer by regulating epithelial-to-mesenchymal transition (EMT)-related genes.^44^ Furthermore, the tumor immune microenvironment (TIME) forms the “soil” for lung cancer development. Specifically, macrophages play a crucial role in the early transformation of lung cancer. The hypomethylated and upregulated *PRAME* can also promote the recruitment of monocytes to tumor tissues and differentiation into tumor-associated macrophages (TAMs), including SPP1 macrophages. Subsequently, SPP1 macrophages secrete migration inhibitory factor (MIF) to inhibit the cytotoxic effect of CD8 + T cells, resulting in immunosuppression. These findings reveal a novel mechanism concerning immune escape of lung cancer.^45,46^ Moreover, we proposed the “epithelium-immunocyte multidimensional cell-cell communication” theory and found that SPP1 macrophages interact with lung stem cells and T cells through SPP1-CD44 and MIF-CD74, thereby maintaining cell stemness and leading to a suppressed TIME, thus promoting the progression of lung cancer (Fig. 2).^45–47^

Novel liquid biopsies have attracted much attention in lung cancer detection benefiting from the less invasiveness and feasible sampling, especially better accuracies than conventional biomarkers.^48,49^ Based on various molecules such as circulating tumor DNA (ctDNA) and cell-free DNA (cfDNA), the liquid biopsy methods have shown potential in lung cancer management from early diagnosis, treatment monitoring, to prognosis prediction.^50–52^ We selected 11 DNA methylation markers, including *CDO1*, *GSHR*, *HOXA11*, etc., and validated their diagnostic abilities of lung cancer through bronchoalveolar lavage fluid (BALF) samples.^53^ Then, to further explore the detection capability of DNA methylation for early-stage lung cancer, two lung cancer alertness by ctDNA methylation (LunaCAM) models, LunaCAM-S and LunaCAM-D, were separately built on 7 and 6 methylation markers, such as *HOXD9* and *SHOX2*, respectively targeting in the discrimination of lung cancer from healthy population and patients with benign pulmonary diseases. The two models finally achieved AUCs of 0.90 and 0.81 for lung cancer screening and diagnosis. In particular, the LunaCAM-S showed stable performance in different stages of lung cancer and detected 86% tumors less than 1.2 cm, which implied that the model may be capable of catching the malignant information in early stage. Moreover, the LunaCAM-D substantially surpassed the CEA test (AUC: 0.56) for diagnosis.^54^

As for non-coding RNAs (ncRNAs), in lung adenocarcinoma (LUAD), the hsa_circ_0072309 (circLIFR) was downregulated and exhibited tumor inhibition function by impressing the cell migration.^55^ Furthermore, we implemented circRNA sequencing in both LUAD and lung squamous cell carcinoma (LUSC), as well as matched normal tissues, then selected four circRNAs of high abundance, namely hsa_circ_0001073, hsa_circ_0001495, hsa_circ_0077837, and hsa_circ_0001821. The hsa_circ_0001073 and hsa_circ_0001495 showed altered expressions in LUAD and LUSC, and the two molecules presented subtyping potential with AUCs of 0.919 and 0.965 for LUAD or LUSC identification.^56^ To further reduce overdiagnosis, it is warranted to establish the validated thresholds to distinguish the indolent lesions from those demonstrate aggressive biological behavior and require immediate intervention.

## Molecular characterization to guide individualized treatment

For resectable early-stage lung cancer, surgical operation serves as the key local treatment.^57^ Nevertheless, for patients with unresectable lung cancer and positive molecular events such as driver gene mutations or immune checkpoint expressions, the treatment has entered the era of precision therapy based on molecular detection; however, there are drawbacks such as invasive procedures and tumor heterogeneity.^58–61^ Thus, to tackle these issues, we explored the non-invasive molecular characterization approaches to guide the optimal therapy scheduling. We utilized whole-lung features on CT scans to predict epidermal growth factor receptor (*EGFR*) mutation, which method reached an AUC of 0.756.^62^ In the same task, another intelligent system was subsequently constructed *via* a self-supervised multitask deep learning strategy, achieving an improved AUC of 0.824.^63^ For discrimination of *EGFR* mutation subtypes, including 19Del, L858R, and others, combination of deep learning features, radiomics features, and clinical features obtained an AUC of 0.841 in this three-way classification task. And the heatmaps highlighted the radiology foundations behind the final outputs.^64^ Furthermore, we quantitatively visualized the activation levels of EGFR in lung cancer patients through a specific molecular probe, HX103, to accurately identify the cohort who are likely to benefit from EGFR-tyrosine kinase inhibitor (EGFR-TKI) targeted therapy.^65^ In addition, to overcome the challenge of EGFR-TKI resistance, we adopted next-generation sequencing (NGS)-based liquid biopsy to identify patients with T790M relative allele frequency (RAF) < 20% who were more likely to develop resistance after the first-generation EGFR-TKI treatment, providing a potential strategy for therapy planning and prognosis prediction.^66^ On the basis of the yes-associated protein (YAP) which is confirmed as a critical driver of EGFR-TKI resistance and the emerging photodynamic therapy (PDT), we proposed a nanococktail therapy implemented by conjugates that were able to deliver gefitinib, YAP-siRNA, and a photosensitizer (Ppa) simultaneously, thus promoting apoptosis of cancer cells and showing a tumor inhibition rate of 86.7%.^67^ Moreover, in terms of another driver gene of lung cancer, anaplastic lymphoma kinase (*ALK*), a capture-based targeted sequencing panel was applied to detect the rearrangement events in plasma, reaching a sensitivity of 79.2%, assisting more precise targeted treatment.^68^

Besides targeted therapy, for immunotherapy, a programmed death-ligand 1 (PD-L1) expression signature (PD-L1ES) was constructed by a deep convolutional neural network to predict the high PD-L1 expression (≥ 50%) by decoding the pretreatment CT volumes.^69^ Then, the combination of deep learning, radiomics, and clinical features predicted the different expressions of PD-L1, namely, <1%, 1-49%, and ≥ 50%, with AUCs of 0.950, 0.934, and 0.946.^70^ Furthermore, a multi-molecular assessment model was further developed to predict multiple events such as *EGFR* and Kirsten rat sarcoma viral oncogene homolog (*KRAS*) mutations, as well as PD-L1 expression, better aligning with the real-world clinical application scenarios and enabling the appropriate therapy planning.^71,72^ Therefore, we defined the “medical imaging ecology” which refers to mining the radiological characteristics of the lesions and surrounded environment and subsequently predicting the biological events, as well as highlighting the interpretability to visualize the correlation between the macroscopic manifestation and underlying microscopic information. Therefore, candidates who may benefit from specific molecule-based treatment can be pre-emptively selected to better assist clinical decision-making.

## Application of China Protocol for early diagnosis and precise treatment of lung cancer

Through the continuous effort, we established the China Protocol, a complete pipeline containing screening, diagnosis, treatment, along with the whole-process management of lung cancer. It is especially suitable for people over 40 years old who are at high risk of lung cancer and are detected with pulmonary nodules on CT scans. Specifically, the high-risk population can be identified *via* estimation of the risk factors; then, Tre-LDCT is able to detect the existing pulmonary nodules from this group by CT examination. Once the nodules are found, different risk levels are determined using C-Lung-RADS. In addition, through individual-specific follow-up based on the trait evolution of pulmonary nodules, lung cancer can be identified as early as possible. Moreover, for the uncertain nodules that are challenging to diagnose by CT images, the novel biomarkers are capable of further improving diagnostic accuracy through combined analysis of the laboratory results and AI predictions. After lung cancer diagnosis, the non-invasive molecular characterization methods are utilized to guide individualized therapy.

**Fig. 3:**
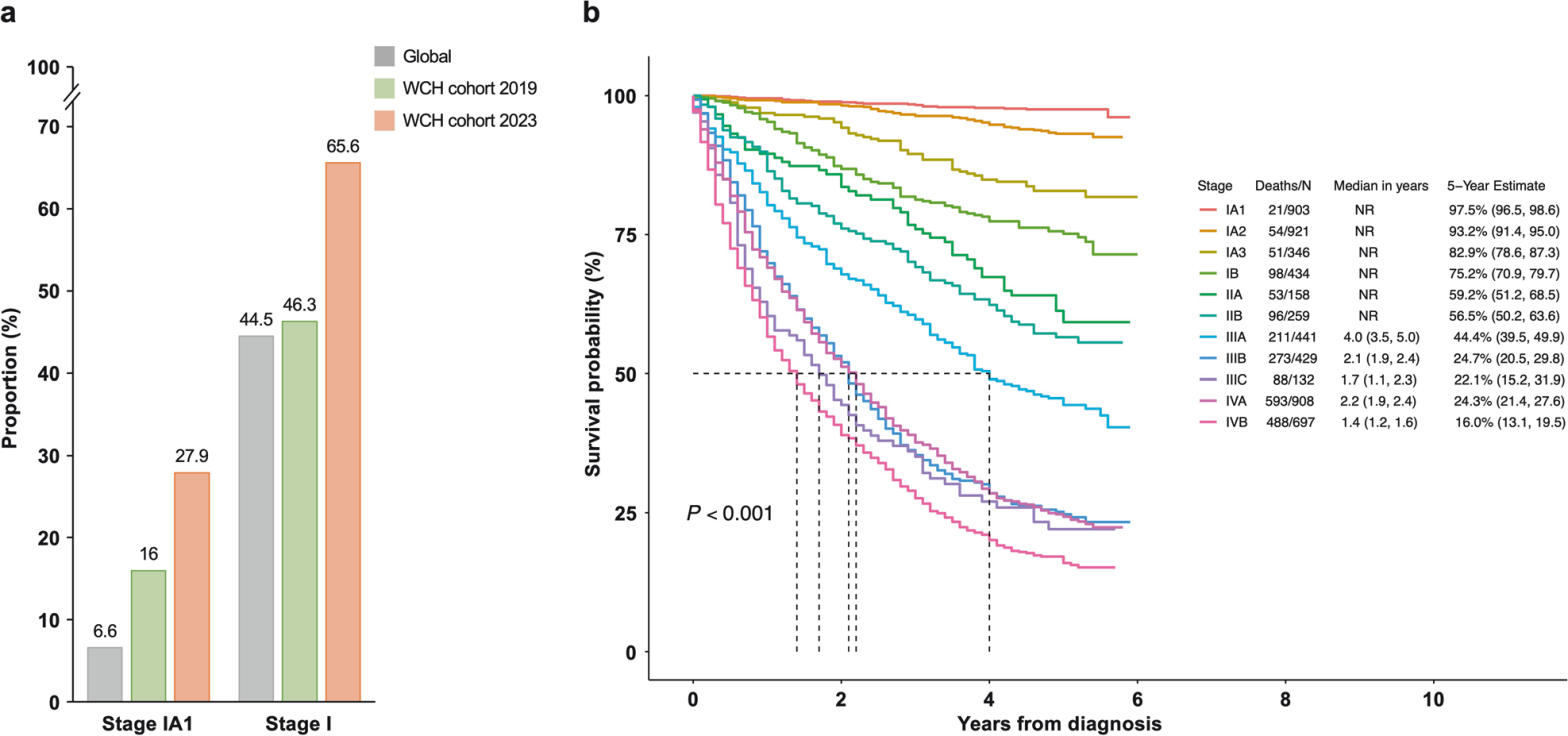
Application results of China Protocol in early diagnosis and survival improvement of lung cancer. **a** Proportion of stage IA1 and stage I lung cancer globally and in West China Hospital of Sichuan University (WCH) cohorts; **b** Kaplan-Meier survival curves of patients diagnosed with lung cancer in 2019. NR, not reached

The implementation of this protocol improved the early diagnosis and 5-year survival rates of lung cancer. And to detailedly quantify the application effect, we enrolled a total of 11,844 patients pathologically confirmed with lung cancer in 2019 and 2023 as examples (Supplementary Table 1). For patients diagnosed in 2019, follow-up data were obtained until December 2024 and the median follow-up time was 46.8 months (interquartile range [IQR], 12-60 months). Detailed clinical characteristics of this cohort were provided in Supplementary Table 2. As a result, the detection rate of early-stage lung cancer achieved 46.3% in 2019 and 65.6% in 2023, surpassing the global proportion of 44.5%. In ultra-early stage lung cancer, this rate has increased from 16% to 27.9%, higher than the international ratio of 6.6% (Fig. 3a).^3^ As for survival, the timely diagnosis has improved the 5-year survival rate of lung cancer to 59%. For early-stage and ultra-early stage lung cancer, the rates reached 90.4% and 97.5%, respectively (Supplementary Table 3, Fig. 3b).

For further application and promotion, our dynamic database of patients with lung cancer not only provides solid data support for the continuous optimization of China Protocol but also enhances the generalization of this strategy in various populations.^30,35^ Furthermore, the wide coverage and efficient implementation of China Protocol are facilitated by the application of advanced technologies, such as mobile CT, 5G communication technology, and Internet of Things (IoT). For instance, in resource-limited areas, patients can undergo lung cancer screening through vehicle-mounted CT and receive diagnosis from experts at tertiary hospitals through cloud computing platforms, improving the accessibility of China Protocol and providing patients in remote locations with equal access to advanced healthcare services.^73,74^ And we have developed the intelligent product for lung cancer diagnosis, which can be applied in different medical institutions once assembled. Moreover, the biomarkers we developed can be detected by low-cost quantitative polymerase chain reaction (qPCR) assay which is practicable and convenient. These advantages make it possible to generalize this protocol widely.

Compared to other lung cancer management strategies, China Protocol was established on a large-scale database, and we redefined the high-risk population based on Chinese cohort and developed the novel Tre-LDCT technology to realize accurate stepwise screening. Then, we innovated AI algorithms for image processing and diverse feature fusion, as well as mining novel biomarkers, for precise diagnosis. Moreover, we explored non-invasive molecular characterization methods to guide the individual-specific treatment, more advanced and convenient than traditional approaches. Despite certain achievements that have been made, there are several limitations of this research. Since participants involved in our investigation were primarily from western China, prospective multi-ethnic multi-center validation is needed. Then, intelligent diagnosis based on the multi-dimensional fusion of medical images and molecules to generate more accurate clinical decisions is worthy of deep research. In addition, as the LLMs have become the frontier of AI, developing generative models is useful to enable human-computer interactive clinical workflow of lung cancer.

In summary, China Protocol has yielded achievements in early screening, precise diagnosis, and individualized treatment of lung cancer, opening a new chapter for this disease management. In the future, more technological innovation and strategy optimization suitable for real-world settings are promising to revolutionize the clinical paradigm of lung cancer, thus improving the patient care.

## Supplementary information

Supplementary Tables

## Data Availability

All the data are available from the corresponding authors upon reasonable request.
