# Supplementary material for: China Protocol for early screening, precise diagnosis, and individualized treatment of lung cancer
Source: Signal Transduct Target Ther. 2025 May 27;10:175. doi: 10.1038/s41392-025-02256-1 (PMC12117065; doi:10.1038/s41392-025-02256-1)
Supplement: Supplementary file 1 — Supplementary Tables [file 41392_2025_2256_MOESM1_ESM.docx]

Supplementary Materials for

**China Protocol for early screening, precise diagnosis, and individualized treatment of lung cancer**

Chengdi Wang^1,2 🖂^, Bojiang Chen^1^, Shufan Liang^1^, Jun Shao^1^, Jingwei Li^1^, Liuqing Yang^1^, Pengwei Ren^1^, Zhoufeng Wang^1^, Wenxin Luo^1^, Li Zhang^1^, Dan Liu^1^, Weimin Li^1,2 🖂^

Correspondence to: [weimi003@scu.edu.cn](mailto:weimi003@scu.edu.cn); [chengdi_wang@scu.edu.cn](mailto:chengdi_wang@scu.edu.cn)

**This document includes:** Supplementary Tables 1-3.

**Supplementary Table 1. Clinical characteristics of the included patients with lung cancer**

| **Characteristics** | **2019 (*N* = 5,628)** | **2023 (*N* = 6,216)** |
| --- | --- | --- |
| **Age** — yr |  |  |
| Median | 60 | 58 |
| Interquartile range | 52-67 | 52-66 |
| **Sex** — no. (%) |  |  |
| Male | 3,036 (53.9) | 2,598 (41.8) |
| Female | 2,592 (46.1) | 3,618 (58.2) |
| **Stage** — no. (%) |  |  |
| I | 2,604 (46.3) | 4,078 (65.6) |
| IA1 | 903 (16) | 1,733 (27.9) |
| IA2 | 921 (16.4) | 1,463 (23.5) |
| IA3 | 346 (6.1) | 380 (6.1) |
| IB | 434 (7.7) | 502 (8.1) |
| II | 417 (7.4) | 354 (5.7) |
| IIA | 158 (2.8) | 117 (1.9) |
| IIB | 259 (4.6) | 237 (3.8) |
| III | 1,002 (17.8) | 745 (12) |
| IIIA | 441 (7.8) | 327 (5.3) |
| IIIB | 429 (7.6) | 318 (5.1) |
| IIIC | 132 (2.3) | 100 (1.6) |
| IV | 1,605 (28.5) | 1,039 (16.7) |
| IVA | 908 (16.1) | 531 (8.5) |
| IVB | 697 (12.4) | 508 (8.2) |

**Supplementary Table 2. Detailed information of the patients diagnosed with lung cancer in 2019**

| **Characteristics** | ***N* = 5,628** |
| --- | --- |
| **Smoking history** — no. (%) |  |
| Ever | 1,839 (32.7) |
| Never | 3,789 (67.3) |
| **Family history of tumor** — no. (%) |  |
| Yes | 739 (13.1) |
| No | 4,889 (86.9) |
| **Family history of lung cancer** — no. (%) |  |
| Yes | 307 (5.5) |
| No | 5,321 (94.5) |
| **Histological subtypes** — no. (%) |  |
| LUAD | 4,190 (74.4) |
| LUSC | 787 (14) |
| SCLC | 297 (5.3) |
| Others | 354 (6.3) |
| **Surgical** — no. (%) |  |
| Yes | 3,178 (56.5) |
| No | 2,450 (43.5) |
| **Gene mutations** — no. (%) |  |
| Available | 1,607 (28.6) |
| *EGFR* | 887 (15.8) |
| *ALK* | 85 (1.5) |
| *KRAS* | 130 (2.3) |
| *TP53* | 614 (10.9) |
| Others | 793 (14.1) |
| NA | 4,021 (71.4) |
| **Outcome** — no. (%) |  |
| Survival | 2,466 (43.8) |
| Death | 2,026 (36) |
| Lost follow-up | 1,136 (20.2) |

*ALK*, anaplastic lymphoma kinase; *EGFR*, epidermal growth factor receptor; *KRAS*, Kirsten rat sarcoma viral oncogene homolog; LUAD, lung adenocarcinoma; LUSC, lung squamous cell carcinoma; NA, not available; SCLC, small cell lung cancer.

**Supplementary Table 3. 5-year survival rates of the** **patients diagnosed with lung cancer in 2019**

| **Stage** | **5-year estimate** |
| --- | --- |
| I — % of patients (95% CI) | 90.4 (89.2, 91.6) |
| IA1 | 97.5 (96.5, 98.6) |
| IA2 | 93.2 (91.4, 95) |
| IA3 | 82.9 (78.6, 87.3) |
| IB | 75.2 (70.9, 79.7) |
| II — % of patients (95% CI) | 57.4 (52.4, 63) |
| IIA | 59.2 (51.2, 68.5) |
| IIB | 56.5 (50.2, 63.6) |
| III — % of patients (95% CI) | 33.2 (30.1, 36.7) |
| IIIA | 44.4 (39.5, 49.9) |
| IIIB | 24.7 (20.5, 29.8) |
| IIIC | 22.1 (15.2, 31.9) |
| IV — % of patients (95% CI) | 20.7 (18.6, 23.1) |
| IVA | 24.3 (21.4, 27.6) |
| IVB | 16 (13.1, 19.5) |

CI, confidence interval.
